# The Quest for Psychiatric Advancement through Theory, beyond Serendipity

**DOI:** 10.3390/brainsci12010072

**Published:** 2021-12-31

**Authors:** Robert E. Kelly, Anthony O. Ahmed, Matthew J. Hoptman, Anika F. Alix, George S. Alexopoulos

**Affiliations:** 1 Department of Psychiatry, Weill Cornell Medicine, White Plains, NY 10605, USA; aoa9001@med.cornell.edu (A.O.A.); gsalexop@med.cornell.edu (G.S.A.); 2 Clinical Research Division, Nathan S. Kline Institute for Psychiatric Research, Orangeburg, NY 10962, USA; matthew.hoptman@nki.rfmh.org; 3 Department of Psychiatry, New York University Grossman School of Medicine, New York, NY 10016, USA; 4 Teachers College, Columbia University, New York, NY 10027, USA; afa2129@tc.columbia.edu

**Keywords:** mind–brain gap, research methods, metascience, psychiatry, medical model, precision medicine, Research Domain Criteria (RDoC), Diagnostic and Statistical Manual of Mental Disorders (DSM), International Classification of Diseases (ICD)

## Abstract

Over the past century, advancements in psychiatric treatments have freed countless individuals from the burden of life-long, incapacitating mental illness. These treatments have largely been discovered by chance. Theory has driven advancement in the natural sciences and other branches of medicine, but psychiatry remains a field in its “infancy”. The targets for healing in psychiatry lie within the realm of the mind’s subjective experience and thought, which we cannot yet describe in terms of their biological underpinnings in the brain. Our technology is sufficiently advanced to study brain neurons and their interactions on an electrophysiological and molecular level, but we cannot say how these form a single feeling or thought. While psychiatry waits for its “Copernican Revolution”, we continue the work in developing theories and associated experiments based on our existing diagnostic systems, for example, the Diagnostic and Statistical Manual of Mental Disorders (DSM), International Classification of Diseases (ICD), or the more newly introduced Research Domain Criteria (RDoC) framework. Understanding the subjective reality of the mind in biological terms would doubtless lead to huge advances in psychiatry, as well as to ethical dilemmas, from which we are spared for the time being.

“That’s my reflection I see in the mirror, isn’t it?” Betty asked, seeking confirmation from her psychiatrist. Until then, she believed that she saw her “evil twin sister” in the mirror, the last of many tormenting paranoid delusions fading into distant memories. Betty also realized that the antipsychotic medication that she had been taking for the previous six weeks caused this remarkable improvement in her mental health. She soon returned home to family and friends, resumed her previous career, and got back to her life as usual, as if the past year and a half had been nothing more than a bad dream. In her mind’s eye, Betty could see the world clearly once again. Although her schizophrenia was not cured, with one pill a day, it was remedied as easily as myopia with a pair of eyeglasses.

Betty’s is one of countless stories to which psychiatrists are privileged to bear witness and serve as catalysts for life-altering, miraculous change. Psychiatrists and their patients today enjoy the fruits of amazing discoveries, which have facilitated dramatic reductions in the “insane asylums” of the past century [1,2]. These advances in the field of biological psychiatry have not been guided by a Copernicus or an Einstein developing theories about the universe from first principles, but by inaccurate or incomplete theories driving experiments, which luckily have led to important empirical observations.

For example, the first antipsychotic medication was discovered in 1952 at St Anne’s Hospital in Paris, when Jean Delay and Pierre Deniker tested an anesthetic used in the operating theater (chlorpromazine) to see if it might help patients with agitated psychotic mania by inducing “artificial hibernation” [2,3]. Electroconvulsive therapy (ECT), the gold standard for treatment of severe depression, was initially trialed in Italy as a treatment for schizophrenia in the 1930s by Ugo Cerletti and Lucio Bini, based on the later unsupported theory that epilepsy and schizophrenia are mutually antagonistic [4]. In 1949, while working with the hypothesis that uric acid toxicity was involved in bipolar mania, Australian psychiatrist John Cade observed that lithium urate appeared to have a calming effect on guinea pigs, leading to the discovery of lithium as an effective treatment for bipolar disorder [5].

Although serendipity could continue to serve as the main driver of psychiatric research, advancements in the natural sciences and other branches of medicine show the power of theory-driven development. Consider recent advancements within the field of infectious diseases in understanding and addressing the AIDS epidemic [6] as well as to contain two deadly worldwide coronavirus epidemics and to reduce mortality from COVID-19 [7]. Unfortunately, despite significant advances in understanding cell function at a molecular level and the availability of a host of psychotropic medications, our theories about how these medications work are often challenged by contradictory evidence. As an example, while nearly all antipsychotic medications are dopamine antagonists, the most effective one, clozapine, only weakly blocks dopamine receptors, contradicting the theory that dopamine receptor antagonism alone causes the antipsychotic effect [8,9,10].

The enormous gap between the mind and brain remains. No one has yet figured out how to describe subjective experience and thought within the realm of the mind in terms of its biological underpinnings in the brain. We can study and catalog interactions among brain neurons but cannot say how these form a single thought. Depression is a leading cause of disability worldwide, yet exactly what this corresponds to in the brain remains unknown [11].

How do we move from our reliance on serendipity to scientific discovery through theory? Theories serve as instruments of action by providing the framework for designing experiments aimed to challenge their assertions. We entertain theories as long as attempts to falsify them have failed [12]; however, multiple theories can often explain the same experimental results [13]. Although it may not be possible to ascertain the “truth” of any theory, the value in our theories lies in their explanatory power and ability to reliably predict relationships among variables, especially causal relationships, which lend the power to change our environments and ourselves for the better [14,15]. Science offers no help in deciding what is better, which must be determined subjectively, influenced by societal norms, but a consensus can often be reached [16]. The determination that cigarette smoking causes lung cancer, among other health problems, has led to successful campaigns to reduce cigarette smoking and its associated health problems [17].

As a field in its infancy, psychiatry can apply the same “medical model” that has been successful in other fields of medicine. For example, physicians were able to diagnose syphilis, with its widely varying manifestations, centuries before successful treatments became available or the spirochete Treponema pallidum could be visualized microscopically [18]. The medical model can be conceptualized as a process of “pattern recognition” that utilizes signs and symptoms to determine the diagnosis, which in turn can help to predict the course of illness and prognosis and guide research to provide insight into the etiology, pathogenesis, and treatment [19]. The Diagnostic and Statistical Manual of Mental Disorders (DSM) and the analogous International Classification of Diseases (ICD) are based on this model [20].

Critics of the DSM (and ICD) approach maintain that it lacks a mechanistic background, with its focus on objective criteria to promote diagnostic agreement among clinicians. In addition, its diagnostic categories, such as Major Depressive Disorder and Schizophrenia, consist of heterogeneous subgroups, which may someday be considered separate disorders, with different underlying etiologies and responses to treatment [21]. Attempts to relate these broad categories to underlying brain biology have been largely unsuccessful [22]. Further advancement in psychiatry might proceed via biomarkers that identify subgroups to be targeted with treatments specific to each subgroup, the way that cancers are now classified according to their genetic signatures to determine the choice of therapies [23].

The need for such “precision medicine” motivated the development of the Research Domain Criteria (RDoC) framework. RDoC focuses on individual brain functions and neural circuitry rather than on the symptom clustering and descriptive nosology that has been the focus of the DSM and ICD systems [23]. Some heterogeneity in studied groups would still exist with the RDoC approach. For example, a study of fear-potentiated startle in individuals with high levels of anxiety would include individuals with a variety of DSM/ICD diagnoses. However, well-separated “biotypes” can be found within or across traditional diagnostic categories, using measures that are not directly related to psychiatric signs or symptoms, such as measures of “cognitive control” and “sensorimotor reactivity” [20,24]. The hope with RDoC is that some of its research domains may relate more directly to behavioral science and underlying neurobiology, providing insights into brain function that would eventually lead to new, successful treatments of psychiatric illnesses [23].

While psychiatry waits for its “microscopes” and “relativity theories”—tools and theories to better elucidate the connection between the mind and brain—much work remains in utilizing our existing frameworks to validate relevant variables, develop reliable measures, and find relationships among them for psychiatric advancement. Common to the RDoC, DSM, and ICD approaches to research is the identification of relevant constructs to describe theorized brain states and functions. These latent or underlying constructs can only be inferred from measurable phenomena, so the work of identifying these constructs, relating them to reliable measures, and validating the measures among the intended populations is essential. For example, the Montreal Cognitive Assessment (MoCA) tests various aspects of cognition and may be considered a screening test of “global cognitive impairment” because it has been validated as a tool that can distinguish between cognitively healthy people and people who have minimal cognitive impairment or Alzheimer’s disease [25,26]. However, if the MoCA were used on a population of exclusively cognitively healthy people, the differences in scores among people in that group could no longer be said to reflect “cognitive impairment” (ceiling effect), and it would be difficult to make inferences regarding the meaning of such differences [27]. The Autism-Spectrum Quotient (AQ) is another example of a measure whose meaning can vary depending upon the study population. Although highly statistically significant group differences in AQ were found between individuals with autism spectrum disorder and normal controls, no differences were found when comparing such individuals with those with schizophrenia spectrum disorders [28].

Understanding the subjective reality of the mind in biological terms would doubtless lead to huge advances in psychiatry, as well as to ethical dilemmas. The constitution of the World Health Organization describes our goal as health care providers to promote “complete physical, mental and social well-being” [29]. People of nearly all religions, political affiliations, and philosophical persuasions can agree on the general concepts of good and bad [15], as well as the value judgments that happiness and well-being are good things and the opposite, pain and suffering, are bad. Psychiatry is the only branch of medicine that directly targets the biological substrates corresponding to these states of mind. Difficult ethical questions would surely emerge if and when our technology becomes sufficiently advanced to reproduce such experiential phenomena in the lab, separate from living beings whose creation according to theory required millions of years of evolution. As frustrating as our ignorance in psychiatry may be, at least for the time being, we are spared from having to confront these dilemmas.

## Data Availability

No human or animal data were used for this article. “Betty’s” story was woven from a tapestry of realistic events that could apply to millions of people, but are not specific to any individual.
